# Impact of High School Quality on Academic Performance Throughout Medical School

**DOI:** 10.7759/cureus.31496

**Published:** 2022-11-14

**Authors:** Lina J Chan, Deepal Patel, Arbab Khalid, Kencie Ely, Gemma Lagasca, Edward Simanton

**Affiliations:** 1 Medical Education, University of Nevada Las Vegas School of Medicine, Las Vegas, USA; 2 Office of Medical Education, University of Nevada Las Vegas, Las Vegas, USA

**Keywords:** correlation, step 1, usmle, secondary education, reading proficiency

## Abstract

Introduction: Numerous studies currently evaluate medical school success and performance using college Grade Point Average (GPA) and Medical College Admissions Test (MCAT) scores. These studies demonstrate that students who score low on the MCAT will continually perform worse than their peers on medical school exams and board exams. We investigated where a student attended high school and how that factor can affect medical school performance because most studies evaluated performance based on college attendance.

Methods: Our retrospective study evaluated 184 students at Kirk Kerkorian School of Medicine (KSOM) and showed higher-quality high schools, in comparison to lower-quality high schools, affected medical school performance. We categorized two groups for high school quality based on the U.S. News scorecard for programs within Nevada: those students with a high school reading proficiency (HSRP) >50% and those with an HSRP <50%. These two groups were then standardized based on percentile within the school and averaged using HSRP, MCAT, pre-clinical, step 1, clinical, and step 2 scores. A line chart was graphed to demonstrate the difference between the two groups.

Data/results: As might be expected, our results showed significantly higher MCAT scores from students who attended high-quality versus low-quality high schools. Our results also showed that although students from low-quality high schools performed worse for the first part of medical school, by step 2, students will score similarly in both groups.

Conclusion: Students who performed poorly on the MCAT and attended lower-quality high schools will score as competitively as their peers by step 2.

## Introduction

Although multiple studies show a correlation between predictive college variables (Medical College Admissions Test [MCAT], undergraduate Grade Point Average [GPA]) and medical school success, little is known about how high school quality may affect medical student performance (United States Medical Licensing Examination [USMLE] step 1, step 2 CK). This dilemma causes one to question whether a predictive model can be used to retrospectively evaluate medical school performance as far back as high school. The purpose of our study is to determine if high school reading proficiency (HSRP), which is at least four years prior to admission into medical school, can still significantly impact medical school performance. The relationship between high school quality and medical school performance may be debated, but assessments could be integrated during admission to understand differences in standardized exam scores. We asked the following questions in our study: (i) What predictive values can be used as far back as high school to predict medical student success? (2) How can we delineate two separate groups based on high school quality to assess performance?

Unlike most other countries, the United States requires a bachelor’s degree before entering medical school. Most graduate programs evaluate undergraduate performance for the prediction of graduate school performance. Because of this process, limited studies exist in the United States that assess correlations between high school performance and graduate school performance.

High school performance is typically evaluated using the GPA in conjunction with the Scholastic Aptitude Test (SAT) or American College Test (ACT). In particular, a study by Al-Mazrou examines the link between high school GPA and medical school performance (as measured by graduation and drop-out rates) at the College of Medicine at King Saud University in Riyadh, Saudi Arabia [1]. While the study concludes that a low high school GPA, which was defined as under 3.75, correlates with poorer outcomes in medical studies, it is difficult to extrapolate these results to student populations in the United States since the study was completed at a medical school where students enter directly after high school rather than attending a four-year university before medical school. Allensworth and Clark examine the correlation between high school GPA and ACT scores and undergraduate graduation rates and find that GPA tends to provide a stronger correlation to graduation than ACT scores [2].

Another commonly used standardized measure of high school academic performance is the SAT score, a composite score reflecting student performance in reading, writing, and mathematics. Due to its standardized nature and single-time point assessment (as opposed to the school-specific and longitudinal GPA metric), factors such as high school ranking and relative school resources heavily influence the SAT and the ACT. High schools with greater resources and funding are hence advantaged over their counterparts in mean student SAT performance. To the best of our knowledge, limited research examines the relationship between SAT scores and graduate school performance. Namely, in Article 7 Volume 80 of St. John’s Law Review journal, Dr. Roy Freedle discusses the inherent biases in standardized tests as they relate to graduate school performance in law school [3]. Further, Davis et al., in the journal of the Association of American Medical Colleges (AAMC), examine interethnic bias in the MCAT exam; while they briefly discuss the SAT exam, no assessment is provided of its link to graduate school performance [4].

Numerous studies evaluate what factors predict a medical student's success during their pre-clinical years. This success involves passing their courses as well as USMLE Step 1. The most common variables assessed are undergraduate GPA and MCAT scores, which are useful predictors in determining the success of an applicant in medical school.

An important aspect of student evaluation is finding a valid, standard measure that establishes equal opportunity and limits test bias for students with limited resources. One unique factor evaluated for medical student success is a diverse student body, which improves teaching, learning, and altruistic behavior in students’ future practices [5-7]. Casey et al. conducted a retrospective study that evaluated 435 students at Mayo Medical School and concluded that early low standardized examination scores (MCAT) were predictive of low standardized examination scores throughout medical school (USMLE 1 and 2, NBME examination scores). This study’s conclusion agrees with previous studies hosted by different institutions, such as the AAMC, and demonstrates a consensus amongst the medical education community showcasing how a low MCAT score is predictive of poor medical school performance [8].

The AAMC has conducted numerous studies over the past few decades evaluating medical student performance in relation to their MCAT score. Socioeconomic status (SES) was evaluated in 2010 using a national average mean MCAT score of 28 as a cut-off point. Evaluation of low and high SES in both groups demonstrates a higher dropout rate within the first two years of medical education in the low SES group, regardless of the MCAT score [9]. A more recent study by the AAMC in 2020 evaluated the 2015 MCAT, which shows a higher predictive value for medical school performance than undergraduate GPA and can strongly predict medical school performance in pre-clerkship courses and USMLE exams [10]. This study also demonstrates that GPA and MCAT can be used together to evaluate a student's long-term success in medical school and their chances of graduating within four years.

A comparative study to ours includes the findings of Davis et al., which showed that individuals from disadvantaged backgrounds, including low SES, disadvantaged race, and underrepresented minorities (URM), score lower than their white counterparts. This study demonstrates that black and Latino students score lower on the MCAT, GRE, LSAT, and other admissions tests [4]. Davis et al. provided evidence that no bias exists within the MCAT against minority groups in medical school performance through a differential prediction analysis. This analysis revealed the same predictive success amongst URM students and their counterparts in medical school based on MCAT scores but did suggest factors that may contribute to lower standardized exam scores, such as parental education, neighborhood, and school conditions. These factors were suggested to potentially limit a student’s academic potential. Currently, no predictive studies explore how parental education or school conditions may impact academic performance. However, the study by Boxer et al. demonstrated a negative relationship between neighborhood crime rate and academic performance [11]. The study by Ruiz et al. found that low SES is related to lower academic performance, which can be related to lower parental education, and also that a violent neighborhood crime rate partially mediated this relationship [12].

The purpose of this study was to compare the academic performance in the medical school of students who graduated from a high-quality high school to that of students who graduated from a low-quality high school. The novelty of our study is that we looked past individual performance and analyzed our data primarily for the high school students who attended our assessment of medical school performance.

## Materials and methods

This retrospective study analyzed data from 184 students who attended the Kirk Kerkorian School of Medicine (KSOM) from 2017 to 2021. We deidentified data for this study from institutional databases under the approved IRB protocol. We obtained UNLV Biomedical IRB approval for this study.

Since KSOM is a state institution, most of this population comes from local high schools or, secondarily, outlying high schools in Nevada. Furthermore, Clark County, from which the vast majority of this sample comes, is known to be educationally challenged [13]. Schools in this district maintain achievement segregation and a lack of student mobilization, especially in low-quality schools, which is concerning when considering the long-term effects on graduate students [13].

The name of each high school was collected for these 184 students as recorded in the American Medical College Application Service (AMCAS). High- versus low-quality ratings of high schools were assigned using each high school’s average reading proficiency level. The reading proficiency levels of the high school from which each student graduated were input on a 0-100% continuous variable scale. This reading proficiency ranking was provided by the US News website (https://www.usnews.com/best-colleges/rankings/national-universities), which ranked high school rankings regionally and nationally as well as a scorecard using math proficiency, reading proficiency, graduation rate, AP classes offered, and AP class pass rate. Of the five variables analyzed, HSRP demonstrated the greatest statistically significant correlation. For purposes of data analysis, a high school reading proficiency level of greater than or equal to 50% (indicating a high-quality high school) was coded as "1," whereas less than 50% reading proficiency (indicating a low-quality high school) was coded as "0." Students in high schools who did not disclose this information, such as out-of-state students or those who attended private high schools, were taken out of the statistical analysis. In this manner, two groups were identified for further descriptive breakdown and unpaired t-tests.

Additionally, group performance over time was calculated as each exam was administered. To determine this calculation, every student who took an examination (including students from both high school quality categories and students for whom no high school quality data were available) was sorted based on ascending performance and normalized into a respective percentile. Once a corresponding percentile for each student was calculated per exam, students were re-sorted into groups by their high school quality. Each group, with its own set of students who now have a calculated percentile for their respective exam, was defined to have a reference value indicative of overall performance by averaging all percentiles found within that group.

## Results

We first determined whether there were differences in examination results between the high school reading proficiency groups. Examinations of interest were: MCAT, the National Board of Medical Examiners tests given in the preclinical phase (preclinical year exams), step 1, the National Board of Medical Examiners tests given in the clinical year (clinical year exams), and step 2. Comparisons of means with statistical significance and standard deviations are shown in Table 1.

**Table 1:** Performances by groups

Exam	High school quality	N	Mean	Std. deviation	P-value
MCAT	Low-quality	80	508.24	4.755	0.025
	High-quality	104	509.86	4.838	
Preclinical year exams	Low-quality	60	82.0%	0.049	0.372
	High-quality	77	82.7%	0.048	
Step 1	Low-quality	57	228.16	15.370	0.227
	High-quality	75	231.40	15.044	
Clinical year exams	Low-quality	37	77.50%	5.698	0.212
	High-quality	45	79.14%	6.012	
Step 2	Low-quality	34	247.59	11.330	0.925
	High-quality	43	247.86	13.403

Statistically significant differences in average exam performance between the high-quality and low-quality school-attended groups were only found for the MCAT (508.24 vs 509.86, p=0.025). While performance between groups was not statistically different during the first part of medical school, the low-quality high school graduates appeared to perform slightly less than their high-quality high school counterparts until step 2.

**Figure 1:**
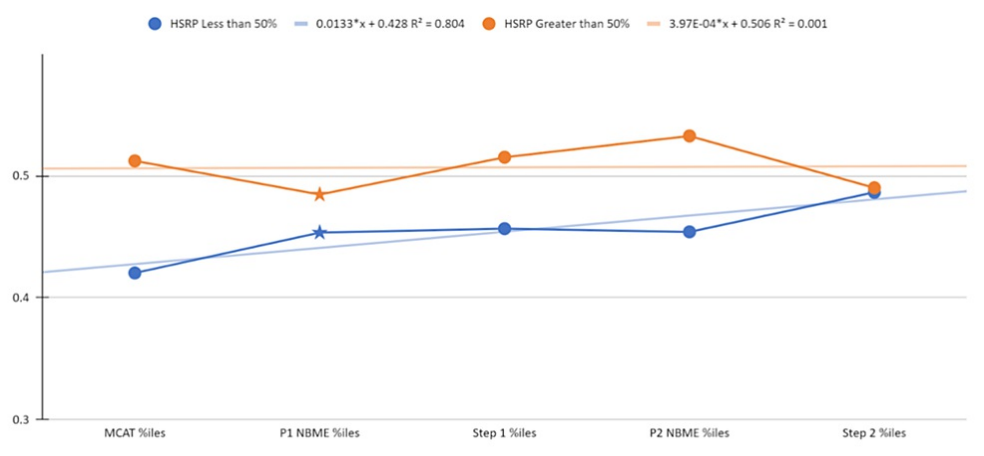
Exam performance trends by high school quality

We then aimed to assess whether both groups (high school reading proficiency less than 50% and high school reading proficiency greater than 50%) remain disparate in exam performance over time or if high school reading proficiency ceases to be predictive of exam performance. To determine this, every student who took an examination, regardless of high school reading proficiency, was sorted based on ascending performance, then normalized into percentiles. Once a corresponding percentile for each student was calculated per exam, students were sorted into groups by their high school reading proficiencies. Student percentiles within each group were averaged. The results are depicted in Figure 1 and Table 2.

**Table 2:** Exam performance trends by high school quality (numerical)

Percentiles	HSRP less than 50%	HSRP greater than 50%
MCAT	0.420	0.512
P1 NBME	0.453	0.485
Step 1	0.457	0.515
P2 NBME	0.454	0.533
Step 2	0.486	0.490

Referring to Figure 1, it is apparent that along the examination trajectory of medical school, high school quality eventually became less predictive of exam performance by the step 2 benchmark, as both groups converge with an average score of 248 and average percentile of 48.65% and 49.02% (Figure 1). Between groups, coming from a high-quality high school appears to be predictive of higher exam performance for the MCAT, phase 1 NBME exam scores, step 1 scores, and phase 2 exam scores. This fact is especially true for step 1 scores and phase 2 NBME scores, in which the high-quality high school group diverges sharply from the other (Figure 1). Interestingly, some points in the curriculum (the pre-clinical year exams and step 2) show both groups below the 50th percentile. This finding would indicate that students for whom high school data were not available (mostly private schools) outperformed their counterparts from either high- or low-quality public schools.

**Figure 2:**
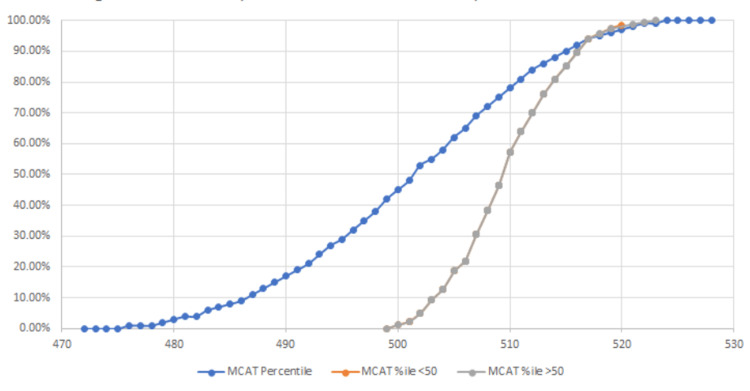
HSRP group performance for MCAT compared to national distribution

**Figure 3:**
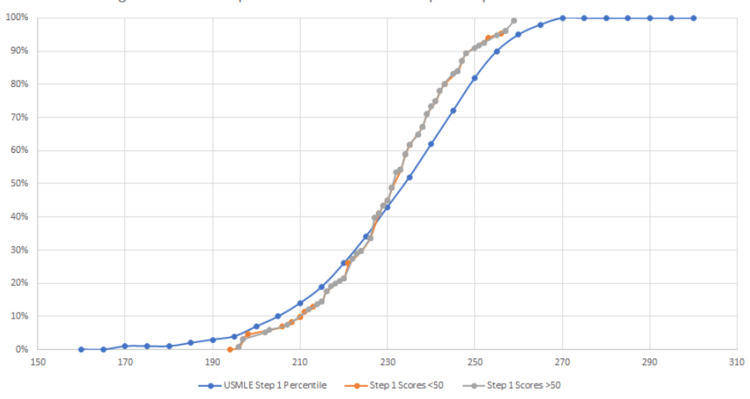
HSRP group performance for USMLE step 1 compared to national distribution

**Figure 4:**
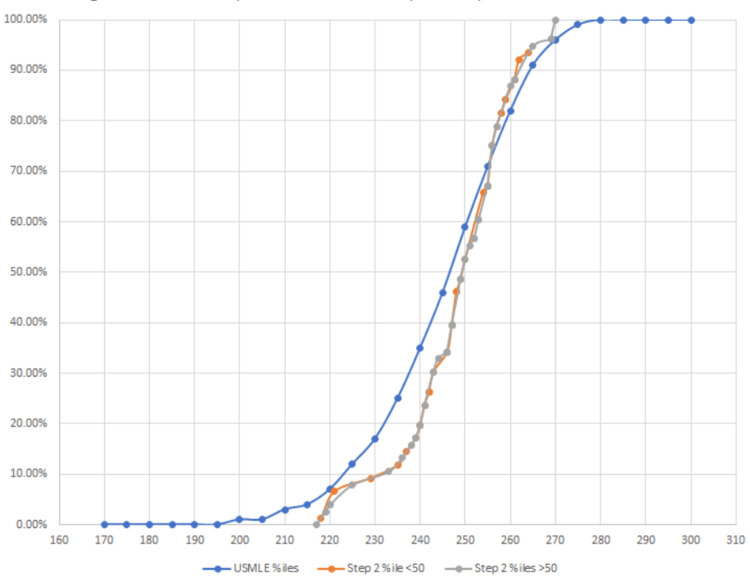
HSRP group performance for step 2 compared to national distribution

It is also necessary to compare each group against national exam distributions in order to determine if there is any favorability for the high school reading proficiency groups for each national board-administered exam (MCAT, steps 1 and 2). In order to assess this, student scores were plotted in a scatter against published data after comparing group averages, as shown in Figures 2-4.

Figure 2 shows that both high school reading proficiency groups (less than 50% and greater than 50%) were completely aligned in their MCAT performance when compared to national percentiles.

**Figure 5:**
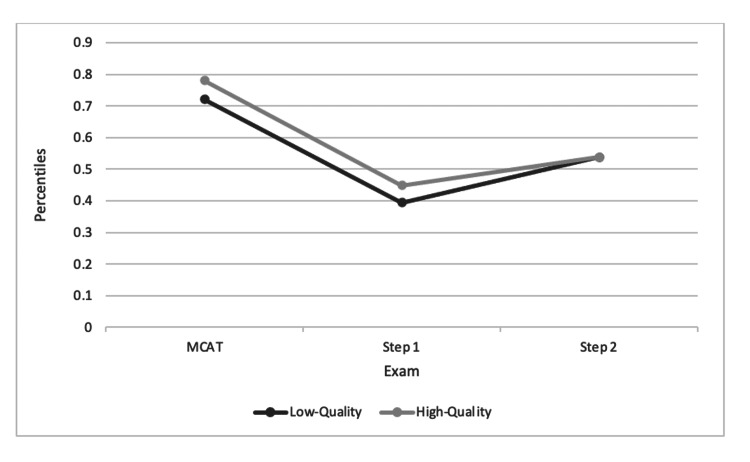
Low-quality and high-quality group exam percentiles standardized to national performance

To provide an external context to our groups, we compared how both groups were performing against the national average scores (Figure 5). For the MCAT, step 1, and step 2 exams, both groups’ exam scores were averaged, rounded to the nearest integer, and compared to the appropriate percentile nationally. For step exams, in which references are only provided for every 5 points, percentiles were interpolated as appropriate.

When assessing performance between low-quality and high-quality HSRP groups (Table 3), there is an evident 6-point difference in performance on the MCAT. By step 1, this 6-point difference becomes a relatively 5-point difference, which still delineates performance between the two groups. By step 2, performance between both groups had converged at 53.8, suggesting homogeneity over time regardless of high school reading proficiency status. During the preclinical years (MCAT, step 1), this gap in performance is common, but it closes during the clinical years (step 2) in our study.

**Table 3:** Low-quality and high-quality group exam percentiles standardized to national performance

Percentiles	Low-quality	High-quality
MCAT	72.0%	78.0%
Step 1	39.4%	44.8%
Step 2	53.8%	53.8%

## Discussion

This study determined that a significant difference exists in MCAT scoring between individuals who attended a high school with a reading proficiency of less than 50%, deemed a "low-quality high school," and those students who attended a high school with a reading proficiency of greater than 50%, deemed a "high-quality high school." Our data analysis used high school quality as a predictive value for performance. Our data show a difference in performance between the two groups, as demonstrated in Figures 1-4, which display a delineation in performance on the MCAT and USMLE step 1. Our data also demonstrate that our analysis of performance within the school population (Figure 1) is also consistent with the national percentile distribution (Figures 2-4). By the step 2 exam, scores converge between the two groups, leaving no obvious disparity between scores. It is also important to note that in our study, the MCAT demonstrated a statistically significant difference in performance even though the average performance between the two groups was numerically close (508 vs. 509; p<0.025). Our results contradict numerous medical school admissions research studies that have suggested MCAT performance as a longitudinal predictive model of medical student success. The divergence in MCAT performance amongst our student body could be due to a lack of resources, as mentioned in previous studies comparing underrepresented minority-status, many of whom attend a less prestigious high school than their counterparts [4,9]. Numerous research studies have shown that high school students with lower resources perform poorer on the SAT and ACT than those who attend a more prestigious school [3,11,12]. This discrepancy may continue contributing to the disparity in standardized scores until the MCAT.

In future studies, we plan to evaluate other high school measures amongst KSOM students in predicting medical student success. Two future measures we plan to study include delineating between first-generation medical students and their counterparts and low-SES students.

Three limitations exist in this study. First, the assessment of group differences decreases with each examination. KSOM is a newer program with a decreasingly limited sample size by step 2. Second, although we can see the separation in group performance when assessing reading proficiency, only the MCAT showed a significant difference compared to the preclinical NBME exams, step 1 clinical NBME exams, and step 2 scores. This separation may also be due to the decreased sample size, and thus an underpowered study when using exams that are tested later in medical school. Third, compared to a traditional medical school, KSOM has a more diverse student body and a higher percentage of underrepresented minority-status students.

## Conclusions

This study found high school quality was predictive of medical student performance on the MCAT. Our study also suggests that students who may score lower on the MCAT will score similarly to their peers on the step 2 examination. We plan to incorporate more predictive values (first-generation, financial aid need) in future studies to strengthen our results. With the transition of the USMLE step 1 score to a pass/fail scoring system, this study may help admissions committees overlook a lower MCAT and factor in a student’s prior education and resources available. We hope that with this approach, students who attended a less privileged high school will be considered in the admissions process with the knowledge that, with the same resources during their clinical years, they will perform similarly.
